# Honeycomb gold specimen supports enabling orthogonal focussed ion beam-milling of elongated cells for cryo-ET

**DOI:** 10.1016/j.jsb.2024.108097

**Published:** 2024-05-19

**Authors:** Victoria L. Hale, James Hooker, Christopher J. Russo, Jan Löwe

**Affiliations:** MRC Laboratory of Molecular Biology, Cambridge CB2 0QH, UK

**Keywords:** Electron cryotomography, Cryo-ET, Lithography, FIB milling, EM grids, FtsZ, Bacterial cytoskeleton

## Abstract

Cryo-focussed ion beam (FIB)-milling is a powerful technique that opens up thick, cellular specimens to high-resolution structural analysis by electron cryotomography (cryo-ET). FIB-milled lamellae can be produced from cells on grids, or cut from thicker, high-pressure frozen specimens. However, these approaches can put geometrical constraints on the specimen that may be unhelpful, particularly when imaging structures within the cell that have a very defined orientation. For example, plunge frozen rod-shaped bacteria orient parallel to the plane of the grid, yet the Z-ring, a filamentous structure of the tubulin-like protein FtsZ and the key organiser of bacterial division, runs around the circumference of the cell such that it is perpendicular to the imaging plane. It is therefore difficult or impractical to image many complete rings with current technologies. To circumvent this problem, we have fabricated monolithic gold specimen supports with a regular array of cylindrical wells in a honeycomb geometry, which trap bacteria in a vertical orientation. These supports, which we call “honeycomb gold discs”, replace standard EM grids and when combined with FIB-milling enable the production of lamellae containing cross-sections through cells. The resulting lamellae are more stable and resistant to breakage and charging than conventional lamellae. The design of the honeycomb discs can be modified according to need and so will also enable cryo-ET and cryo-EM imaging of other specimens in otherwise difficult to obtain orientations.

## Graphical abstract

**Figure:**
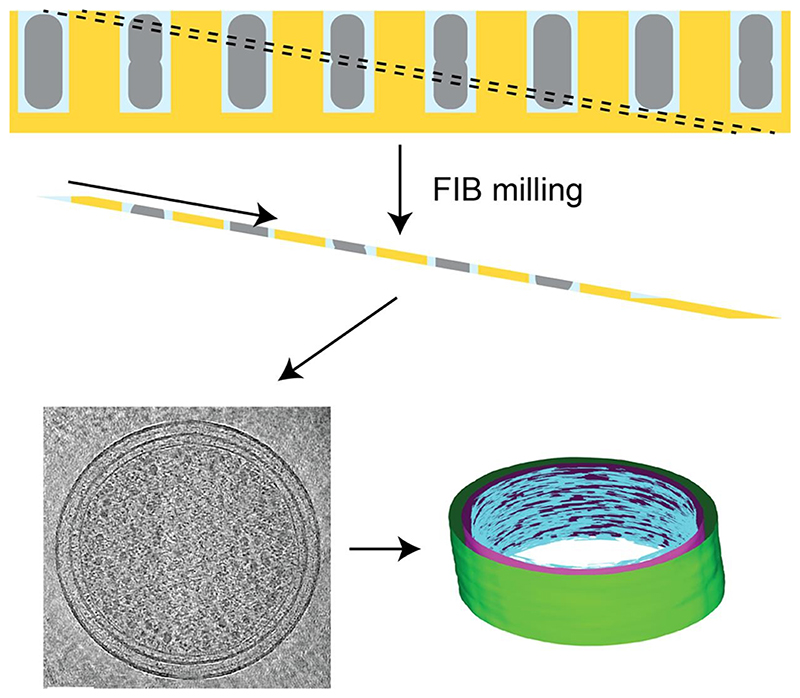

## 1 Introduction

Transmission electron microscopy (TEM) and, in particular, electron cryotomography (cryo-ET) are powerful techniques that allow the visualisation and structure determination of proteins at near-atomic resolutions and also in their native cellular environment. However, the limited mean free path length of electrons in frozen biological materials means that the thickness of the specimen is a key parameter affecting the signal-to-noise ratio of the transmission images collected. To achieve near-atomic resolutions, specimen thicknesses need to be below 200 nm (Dickerson et al., 2022), which is much thinner than most cellular specimens, including most bacteria and archaea. One solution to the problem is cryo-focussed ion beam (FIB)-milling, which allows vitrified cellular material to be thinned into lamellae. This way, lamellae with thicknesses in the tens to hundreds of nanometre range can be produced, enabling the use of cryo-ET for subsequent visualisation and structure determination from specimens that were originally much thicker (Hayles et al., 2010; Marko et al., 2007; Rigort et al., 2012).

A simple and common approach to prepare cellular samples for cryo-FIB milling is plunge freezing on grids, either by culturing adherent cells directly on the grids, or applying cells to the grid. Subsequent blotting removes excess liquid and plunge freezing in liquid ethane vitrifies the sample without allowing the formation of ice crystals. While this approach is technically straightforward and has been successfully used in many studies [for examples see (Hoffmann et al., 2022; Rigort et al., 2012; Wang et al., 2012; Wang et al., 2021)], it has some limitations. Firstly, the thickness is limited to that which can be successfully vitrified by plunge freezing (<10 μm in the best cases, but often < 3 μm) (Dubochet et al., 1988). Secondly, it can lead to problems with preferred orientation of the specimen since each cell’s orientation on the grid will be dictated by the grid’s geometry and by the way the sample falls on the grid. For example, rod-shaped bacteria will tend to lie flat on the grid. This becomes problematic when studying biological features which also have a specific orientation within the cell. Approaches such as the waffle method (Kelley et al., 2022, Klykov et al., 2022) or lift out/serial lift out (Mahamid et al., 2015; Rubino et al., 2012; Schaffer et al., 2019; Schiøtz et al. (2023)) can be used to mill thicker, high-pressure frozen samples. While these methods remove the geometrical constraints of freezing on grids, e.g. high pressure frozen rod-shaped bacteria can be more randomly oriented, they do not allow targeting of specific orientations. This is a problem, for example, when investigating the intracellular structures that control bacterial cell division, since the division plane in most bacteria is precisely orthogonal to the long axis of the cell.

**Fig. 1:**
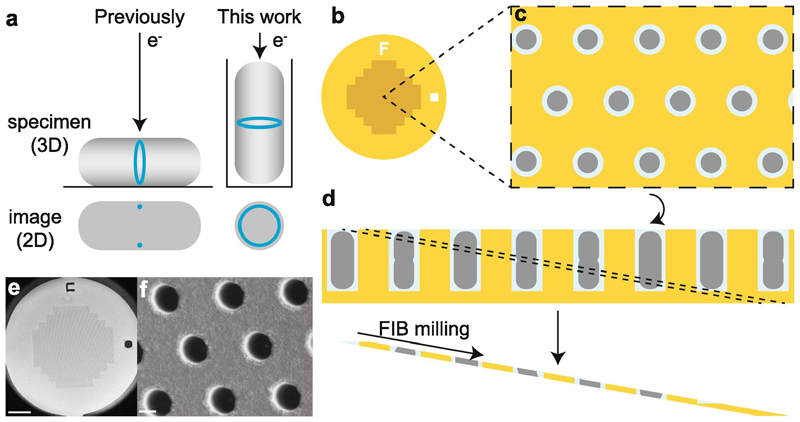
Design of the custom supports (EM discs). (a) Illustration of the effect of cell orientation on imaging the Z-ring in bacterial cell division. (b-d) Cartoon schematics of the custom gold support. (b) Overview of the support. The central region consists of a continuous array of wells to trap bacteria, as depicted face on in (c) and as a side view in (d). The support is FIB milled at approximately 10° with respect to the surface, to create a lamella that samples through the wells at different heights from the front to the back of the disc (dashed lines in [d]). (e, f) SEM images of the custom supports before addition of bacteria, at magnifications to show the whole grid (e) and a small portion of the wells (f), corresponding to the views depicted in (b) and (c), respectively. Scale bars 500 μm (e), 1 μm (f).

The vast majority of archaea and bacteria utilise the protein FtsZ to organise cell division (Barrows and Goley, 2021; Du and Lutkenhaus, 2019). FtsZ is a tubulin homologue that forms filaments around the circumference of the cell at the division site, making a structure known as the Z-ring (Bi and Lutkenhaus, 1991; Li et al., 2007; Wang and Lutkenhaus, 1993). Although there have been several studies using both light microscopy and cryo-ET, there remain fundamental unanswered questions about the nature of the ring *in vivo*, especially at the nanometre scale. While fluorescence microscopy indicates that the ring is likely comprised of short, dynamic filaments (Bisson-Filho et al., 2017; Whitley et al., 2021; Yang et al., 2017), cryo-ET from different groups has suggested that the ring could be formed of continuous or at least overlapping filaments (Khanna et al., 2021; Szwedziak et al., 2014). However, it has never been possible to image complete rings via cryo-ET. This is due to the aforementioned technical limitation of the method, the preferred sample orientation in combination with the missing wedge limitation of cryo-ET. When rod-shaped cells are plunge frozen on EM grids, they orient parallel to the plane of the grid. As the Z-ring runs around the circumference of the cell, this results in images where the Z-ring is only visible in cross section and filaments of FtsZ appear as dots under the inner membrane (Fig. 1a, left). In order to visualise the intact ring, the cell must be oriented vertically (Fig. 1a, right). 3D volumes can be tomographically reconstructed from a series of tilted images, but sample thickness and most microscope hardware limit sample tilting to ± 60–70°. This results in a missing wedge of information, unless the sample is tilted closer to ± 90° (Palmer and Löwe, 2014).

It is important to point out that this problem of cell orientation for visualising the Z-ring is not restricted to cryo-ET studies — it is also a limitation for fluorescence light microscopy. In order to overcome this, an approach has been developed to vertically trap *Bacillus subtilis* in agarose gel micro-holes, allowing the Z-ring to be imaged top-down (Whitley et al., 2022). While that technology could not be adapted to make EM supports, other recent work has shown that standard lithographic techniques can be used to manufacture specimen supports for single-particle cryo-EM at the wafer scale (Naydenova and Russo, 2022). In this work, we aimed to exploit such lithographic techniques.

Here we have developed an approach of vertical entrapment analogous to, yet distinctly advantageous over that used for light microscopy (Whitley et al., 2022), involving grid fabrication techniques (Naydenova and Russo, 2022). When combined with FIB-milling, our approach can be used for cryo-ET of cross sections through bacteria, with the future aim of imaging complete Z-rings and division planes. Compared to previous methods, honeycomb discs reduce specimen charging during milling and reduce specimen movement during imaging; they may also be used for other applications that need specific orientations.

## 2 Results

### 2.1 An EM support design to vertically trap and section rod-shaped bacteria

We designed a sample support, which we call a honeycomb disc, to vertically trap bacteria with elongated shapes; in particular, rod-shaped *E. coli* cells. When combined with FIB milling, the honeycomb discs allowed the generation of lamellae containing cross sections through the bacteria at different heights (Fig. 1b – d). Honeycomb discs replace standard EM grids and comprise a ~ 5 μm thick disc of pure gold of 3.05 mm diameter, that is entirely compatible with standard microscopes and grid holders. Instead of grid squares they contain a continuous, regular array of cylindrical wells, which in this particular design are each 1.5 μm in diameter and 3 μm deep from the surface of the disc (Fig. 1e, f). We chose not to use similar techniques and materials as are used for manufacturing the micro-holes for light microscopy studies (Whitley et al., 2022), but instead use gold as the grid material since it is an excellent electrical and thermal conductor and is undamaged by 300 keV electrons. Gold is easy to mill with standard ion beam instruments and furthermore is chemically inert and non-toxic to cells, allowing prolonged incubation and growth of the bacteria once they are trapped in the wells.

### 2.2 Honeycomb disc manufacture

**Fig. 2:**
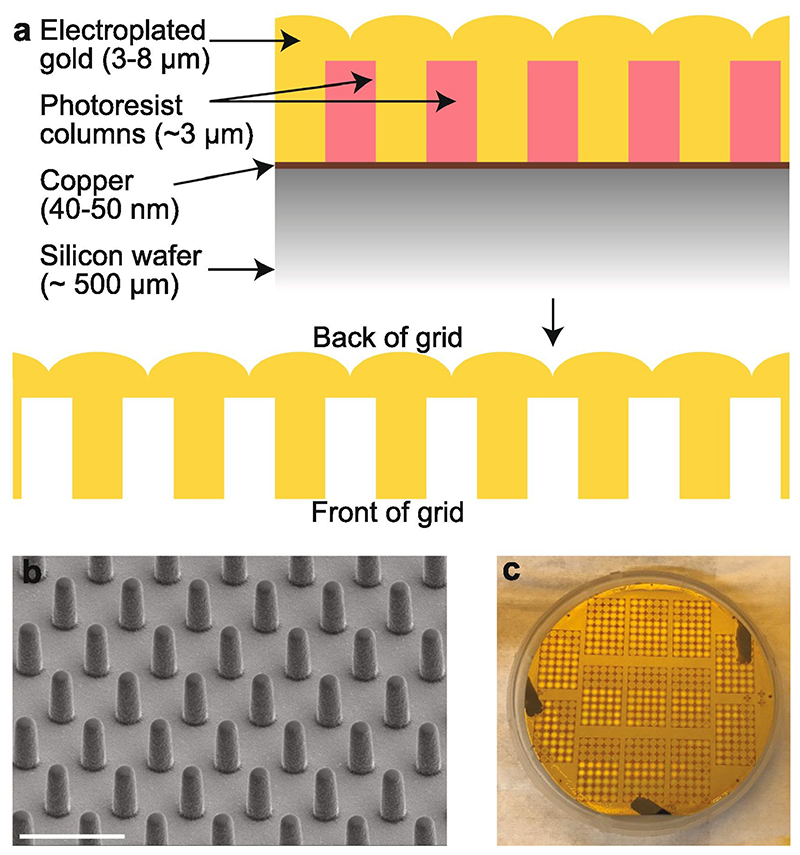
Fabrication of the custom supports. (a) The gold sample supports are fabricated on silicon wafers (grey). First, a thin layer of copper is evaporated onto the wafer which is then patterned with columns of photoresist (pink); SEM image in (b). The patterned wafer is then electroplated with gold to form the wells and individual disc outlines (~400 per wafer), as shown in the photo in (c). Scale bar: 5 µm. Finally, the copper and the photoresist are etched away to release the supports. (For interpretation of the references to colour in this figure legend, the reader is referred to the web version of this article.)

The honeycomb discs were produced in-house using a combination of ultraviolet light lithography and electroforming (Fig. 2a, Materials & Methods). They were manufactured using a 4-inch silicon wafer as a base, yielding approximately 400 discs per wafer. Firstly, a thin (approx. 40–50 nm) film of copper was evaporated onto the wafer using an electron beam evaporator. This acted as both an electrode for the plating step and also as a release layer for the completed discs, but formed no part in the final disc structure. Then, using a lithography mask (Fig. S1), the wafer was patterned with columns of photoresist (Fig. 2b), plus other larger shapes necessary to form the circular outline of the discs and introduce marks to aid with visual identification of orientation when using the discs and to differentiate between different variants of the disc design. Following this, the wafer was electroplated with gold, with the photoresist acting as a mould (Fig. 2c). We found that we needed to plate for an additional depth of a minimum of approximately 1 µm beyond the top of the photoresist to ensure complete gold closure over the photoresist and the fabrication of wells rather than holes. In some instances, we plated up to an additional 5 µm beyond the tops of the photoresist columns. We found that this extra gold significantly slows down the milling process and so the gold layer should be kept to the minimum depth, giving a total disc thickness of around 4 µm including the depth of the wells. Finally, piranha solution was used to etch away the copper and the photoresist, releasing the gold film from the wafer and leaving nothing but the electroformed gold discs remaining (Fig. 2a).

While in the example shown the wells were approximately 1.5 µm in diameter and 3 µm in depth (Fig. 2a), in order to accommodate *E. coli*, the design of the disc is adaptable according to requirements, both in theory and in practice. The 2D design can be altered by changing the design of the lithography mask and the thickness of the support can be adjusted by changing the particular photoresist used and/or the spin coating parameters to alter the depth of the photoresist layer, and with it the plated gold. Additionally, for more complex geometries, it should be possible to do multiple rounds of lithography and plating to build up the device in layers.

### 2.3 Focussed ion beam (FIB)-milling to produce lamellae for cryo-ET

Prior to milling, the honeycomb discs were loaded with bacteria by gentle centrifugation using a custom-made device (Fig S2; Materials & Methods) and vitrified by plunge freezing. As there are no grid bars, lamellae could be milled at any position on the disc. The milling did not need to be targeted to individual cells as each lamella contained many wells, the number of which depended on the well diameter and spacing, and the width of the lamellae (e.g. up to 35 wells for a 25 µm wide lamella). Lamellae were milled at a shallow angle to the disc (typically 8-10°) and therefore sampled through different heights in the wells from the top of the wells at the front of the lamella to the bottom of the wells at the back of the lamella (Fig. 1d, 3a, b). Although discs could be pre-screened by light microscopy (correlative light and electron microscopy, CLEM) prior to milling for well occupancy, ice thickness and specific biological features, we did not use a correlative approach here for targeting the milling to specific cells. It is known that ~ 80 % of *E. coli* show Z-rings during their exponential growth phase, so many cells should have FtsZ filaments at mid-cell height (Tsukanov et al., 2011). We found that the occupancy was somewhat variable, ranging from approximately 50 % to near 100 % in the best cases. Additionally, the occupancy was sometimes variable across the disc. The lack of grid bars or other features for correlation in the honeycomb discs made it difficult to target the best areas for milling as our fluorescence screening was carried out in a separate cryo-confocal microscope. The use of in-chamber fluorescence imaging instead would be useful to more easily target milling to those areas of the grid with the highest occupancy (Boltje et al., 2022; Gorelick et al., 2019; Li et al., 2023; Smeets et al., 2022; Yang et al., 2023).

**Fig. 3:**
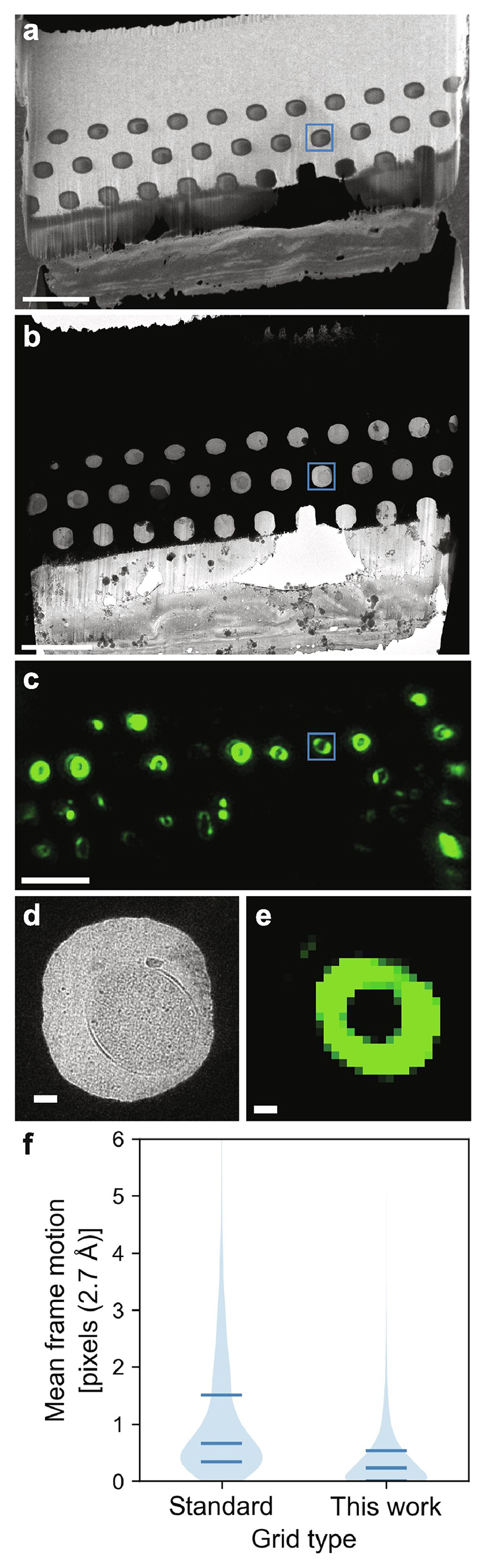
Lamella milling and imaging. A milled lamella was imaged using cryo-SEM (a), cryo-TEM (b) and cryo-confocal microscopy (c). The cryo-confocal image is a projection of the acquired Z stack; the fluorophore is the non-specific membrane dye cellBrite-488. The same cell/well has been indicated in all 3 images (a, b, c). The tomogram in Fig. 5g is from this cell and the cell is shown as seen in the cryo-TEM (d) and as a single slice from the cryo-fluorescence Z-stack (e). (g) Violin plot showing mean motion per frame of tilt series collecting from lamellae milled from the custom support, as compared to convention lamellae. Means: standard grids 3.05 Å, honeycomb discs 1.19 Å after 2.5 e ^−^ /Å^2^ at 300 keV over 6 subframes. Scale bars (a-c): 5 μm; (d, e): 200 nm.

After milling, lamellae can also be imaged by light microscopy, to identify specific targets in the sections. To demonstrate this option, we used a non-specific fluorescent membrane dye that highlights the presence of cell membranes in sections of cells in each well and imaged with cryo-confocal microscopy. We found that the fluorescence signal, in the form of rings, correlated well with the wells where there are visible cells, both in the SEM imaging from the milling and in the medium magnification montage from the TEM (Fig. 3a – e). It is important to point out that in future it will be possible to use this post-milling CLEM step to specifically identify wells containing a target feature/protein and then specifically target those regions for tomography. For example, one could fluorescently label FtsZ or other divisome proteins and then look for sections of cells that contain Z-rings. While doing pilot experiments in this direction, we found that for this to be routinely successful on bacterial cells (approx. 1 µm diameter, sections < 200 nm thick) future advances in the performance of cryo fluorescence microscopy will most likely be needed, and dyes and methods that increase signal from vitrified samples.

Lamellae milled from the honeycomb supports had some advantages in both the FIB-SEM and the TEM, as compared to lamellae consisting of water and biological material alone, as is normally the case. For example, their increased mechanical stability allowed routine milling of lamellae with a width of up to 30 µm. Furthermore, no breakage of lamellae was observed from the handling steps between milling and imaging in the TEM; very occasionally the ice in a small number of wells cracked but this could be avoided by the inclusion of expansion joints, as previously reported for conventional grids (Wolff et al., 2019). Additionally, when compared to conventional lamellae milled through ice/cellular material only, lamellae milled through the honeycomb grids are more stable when being irradiated in the TEM and undergo less motion during tilt series collection (Fig. 3f, violin plot), most likely because the presence of the gold reduces charging during imaging. The aspect ratio of the volume within the well is less than 10, both before and after milling, preventing both buckling during freezing and deformation during electron beam irradiation (Naydenova et al., 2020). Finally, as the gold dominated the milling rate, it helps to protect against curtaining (Fig. 3a, only those wells with little gold in front have significant curtaining). While the honeycomb discs have several key advantages, it is important to note that there are currently some disadvantages. One such disadvantage of the use of gold in the supports resulted in numerous sputtered (redeposited) gold particles on the surface of the lamellae (Fig S3). These produced streaks which extend into the volume of the reconstructed tomograms (Fig S3 a, b), but did allow accurate determination of the location of the two surfaces. Further work is needed to establish methods to better control the gold redeposition during milling. A second disadvantage is the throughput is lower for milling through gold as compared to ice/cellular material alone. In a single day of entirely manual milling, it was possible to mill 2–3 lamellae 25–30 µm wide, which would be equivalent to 4–9 conventional lamellae of a width of 8–12 µm. We anticipate that throughput will be improved using newer instruments/software with more automation (Buckley et al., 2020; Cleeve et al., 2023; Klumpe et al., 2021; Zachs et al., 2020).

### 2.4 Electron cryotomography of FIB-milled gold supports

**Fig. 4:**
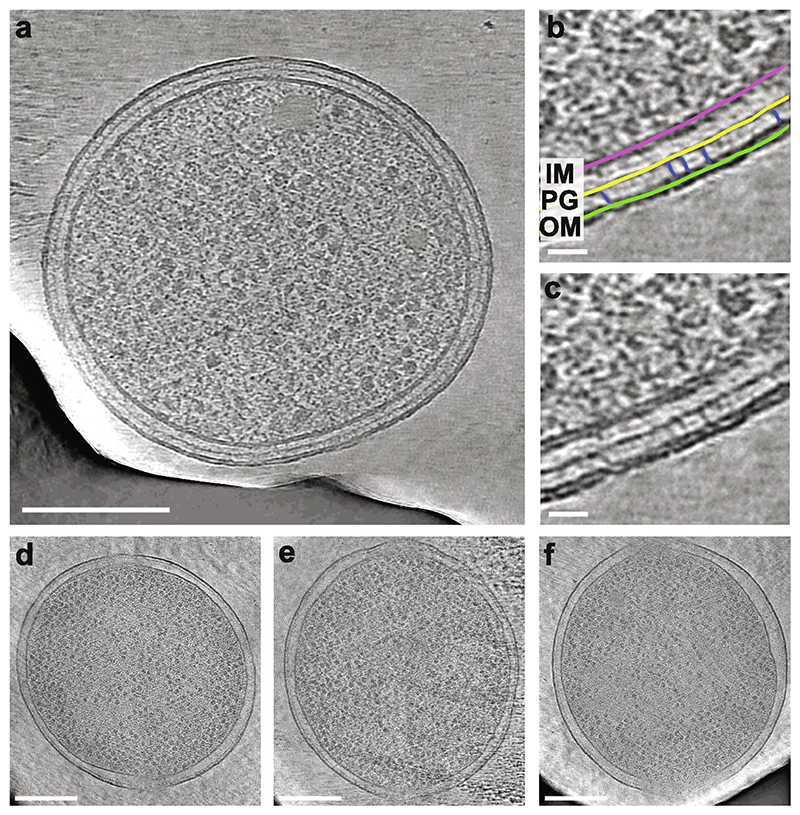
Cryo-ET of *E. coli* bacteria contained in the custom support. (a,d,e,f) Tomogram slices showing cross sections through cells, most likely not at a division plane. The dark regions in the corners are the edges of the gold wells. (b, c) Section of tomogram in (a) depicting the membrane region shown with and without coloured annotation. The inner (magenta) and outer (green) membranes and peptidoglycan (yellow) are visible as well as various protein densities in the periplasm, including those bridging the peptidogylcan to the outer membrane (blue), which could be the very abundant Braun’s lipoprotein (Lpp). Scale bars (a, d, e, f): 250 nm; (b, c): 25 nm. All images shown are a single slice from the tomogram, 4.3 Å thick. (For interpretation of the references to colour in this figure legend, the reader is referred to the web version of this article.)

Lamellae FIB-milled through the wells produce cross-sections that run approximately perpendicular to the long axis of the elongated bacteria (Fig. 1c, d and Fig. 4a, d, e, f). Electron cryo-tomography (cryo-ET) was performed on these lamellae in a transmission electron microscope (TEM), operated at 300 keV. Tilt series were collected with a magnified pixel size of 2.1 or 2.7 Å, depending on data set, which was chosen so that the entire cross-section of a cell fits in the field of view on a ~ 4 k x 4 k pixels image. Tomograms were reconstructed using back projection [IMOD (Kremer et al., 1996)] and subsequently denoised [cryoCARE (Buchholz et al., 2019)]. While it is difficult to measure the resolutions of the resulting tomograms, their quality can be readily estimated from their resolving power of known features of bacterial cells. In the tomograms it is possible to resolve the two leaflets of the membrane bilayers, the peptidoglycan layer, and some protein densities in the periplasm (Fig. 4b, c). In particular, there were a number of stick-like densities bridging between the peptidoglycan and the outer membrane. One of the most highly expressed proteins in *E. coli* is Braun’s lipoprotein (Lpp), a small trimeric coiled coil-containing protein that anchors the peptidoglycan to the outer membrane [reviewed in (Asmar and Collet, 2018)] and is a likely candidate for the bridging densities observed in the tomograms.

To confirm that use of the honeycomb grids results in bacteria that are vertically oriented, we analysed tomograms collected from a single lamella to measure the relative angle of the long cell axis to the lamella. When cells were grown in rich media there was typically a single cell per occupied well, with an average relative angle of 20° over a total range of 6–31 ° (n = 14). In the case where the cell diameter was reduced, for example with FtsZ(D212A) overexpression and cultured in minimal media (see below), we found that rather than observing a high degree of tilt within the wells, instead often multiple cells were present per well. In this case the average tilt was 17°, over a range of 7-21° (n = 9). Decreasing the diameter of the well instead would likely reduce the angular range present. However, the 1.5 µm final well diameter was chosen as we found discs with smaller wells were both more difficult to fabricate and to load with bacteria to an acceptable level of occupancy. Additionally, as the lamellae milling is not perfectly orthogonal to the disc, but is performed at an angle of 8-10° to the grid, some tilt of the cells may prove advantageous. Furthermore, for subtomogram averaging, views from other angles will be required and as such it may prove advantageous to have some tilted cells, although it will most likely still be necessary to combine data collected from the honeycomb discs with those collected from lamellae milled from cells plunge frozen on grids or milled via the waffle method.

**Fig. 5:**
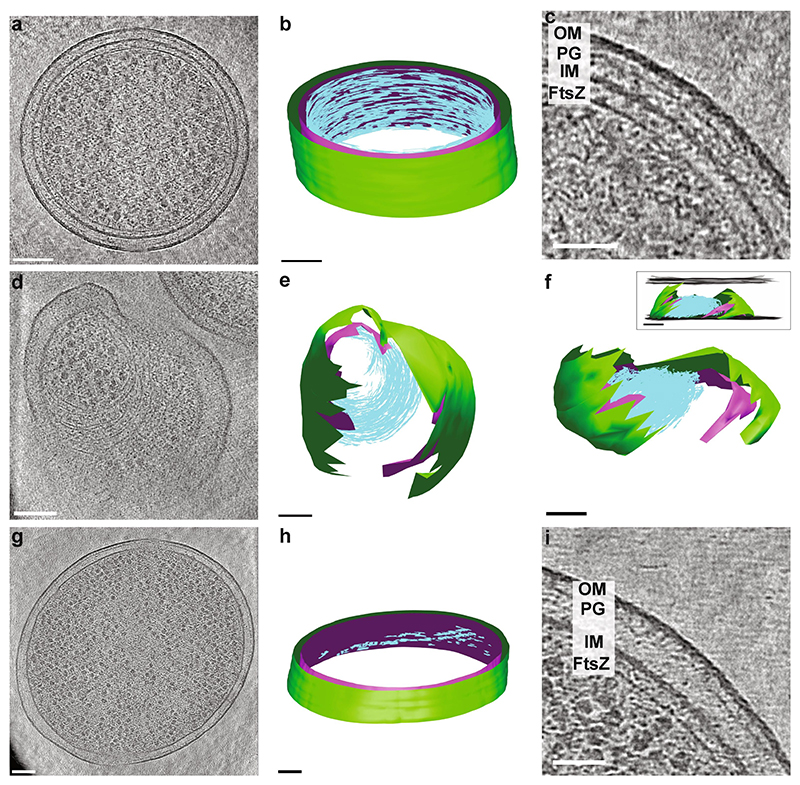
Cryo-ET showing FtsZ filaments. (a) Single slice from a tomogram collected from a lamella of *E. coli* cells over-expressing a GTPase mutant of FtsZ (FtsZ [D212A]). There is an extensive single-layered ring (or band) of filaments (cyan) under the inner membrane (magenta), as modelled in (b). The outer membrane is modelled in green. A close-up of the tomogram is shown in (c). (d – f) FtsZ(D212A) filaments in the overexpression system, this time at a constriction site. This is a cell that sits tilted in the support well. The models in (e) and (f) show top down and tilted side views, respectively. The insert in (f) shows a side view, with the boundaries of the lamella modelled in grey. (g – i) Partial, native Z-ring (no FtsZ mutant or overexpression). Here the plane of the lamella and the plane of the Z-ring ring are not perfectly aligned so that only part of the ring is present in the tomogram. Scale bars (c) and (i): 50 nm, all others 100 nm. Tomogram slice thicknesses (a) and (c) 4.3 Å, (d) (g) and (i) 5.3 Å. OM = outer membrane, PG = peptidoglycan, IM = inner membrane. Note that for the modelling of FtsZ filaments, as it was not always possible to distinguish individual filaments in Z and determine their central slice, contours were drawn on every slice where filaments were visible, so each filament may be represented by multiple contours, rather than a single line. (For interpretation of the references to colour in this figure legend, the reader is referred to the web version of this article.)

In order to increase the chances of observing FtsZ filaments in tomograms, we used overexpression of a previously well-characterised GTPase mutant of FtsZ (FtsZ[D212A]), which hydrolyses GTP more slowly, leading to more and longer filaments at the division site (Redick et al., 2005; Stricker and Erickson, 2003; Szwedziak et al., 2014). In line with that, mid-cell lamellae of cells expressing FtsZ(D212A) resulted in tomograms with many more FtsZ filaments than in the wild-type cells (Fig. 5 a – c). While it was not possible to definitively trace individual FtsZ filaments in all regions of the tomogram depicted in Fig. 5a – c, they formed a very extensive ring structure with a mostly single-layered band of filamentous protein density under the inner membrane, as was observed before (Li et al., 2007; Szwedziak et al., 2014). In the example shown, there was no apparent membrane constriction. However, constriction has been observed in other cells (Fig. 5d – f), as would be expected given that the cells were not synchronised and were present in all cell cycle stages. In the Fig, 5d–f example, the constriction site was not aligned with the plane of the tomogram; instead, the bacterium was tilted inside the well (by approximately 50–60°), rendering large patches of the membrane invisible due to their orientation relative to the missing wedge. Nevertheless, an extensive band of filaments was present, which lies under the inner membrane where this is visible.

In order to image native Z-rings, tilt series were collected and tomograms generated of the same *E. coli* strain without overexpression of FtsZ(D212A). A representative cell is shown in Fig. 5g–I, containing filaments which we presume to be FtsZ based on the proximity to the membrane and similarity in appearance to the overexpressed FtsZ filaments, both in this study and previously published works (Khanna et al., 2021; Szwedziak et al., 2014). The cell was slightly tilted (approx. 22°) with respect to the plane of the lamella and therefore the tomogram did not contain a complete division plane and FtsZ ring. However, a narrow band of FtsZ filaments is visible, showing part of a Z-ring and spanning the entire depth of the tomogram (thickness 150–175 nm). Given the signal-to-noise attainable without further processing of the tomograms and the small size of FtsZ, it was not possible to determine with absolute certainty if the filaments present in the tomogram overlap everywhere, forming a continuous ring or whether there are gaps, as predicted by previous light microscopy studies (Bisson-Filho et al., 2017; Whitley et al., 2022; Yang et al., 2017). Small gaps are possibly present, but large gaps seem to be absent.

### 2.5 Sub-tomogram averaging

**Fig. 6:**
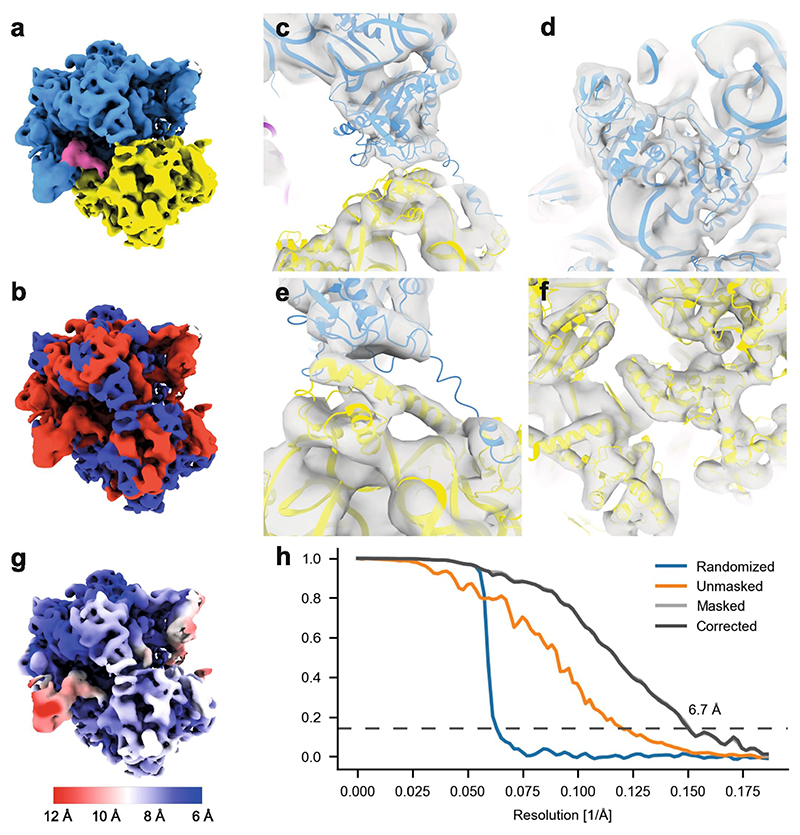
Sub-tomogram average of the 70S ribosome obtained using honeycomb gold supports and FIB-milled cells. (a) The final map, coloured by ribosomal subunit: large subunit in blue, small subunit in yellow, E-site tRNA in pink. (b) The same map, coloured by chemistry: protein in blue, nucleic acid in red. (c–f) Close-up views of the map rendered semi-transparently over PDB model 6H4N, coloured by ribosomal subunit: (c) 50S ribosomal proteins L5 and L31, (d) 50S ribosomal proteins L20 and L21, (e) 30S ribosomal protein S13, (f) 30S ribosomal proteins S2 and S5. (g) The map coloured according to local resolution. Higher resolution (<8 Å) is apparent in the protein components of the large subunit, lower resolution (>10 Å) in the L1 stalk. Images rendered by UCSF ChimeraX (Goddard et al., 2018). (h) Fourier shell correlation (FSC) plot of half-maps generated during refinement by M. The corrected FSC curve crosses 0.143 at 6.7 Å. (For interpretation of the references to colour in this figure legend, the reader is referred to the web version of this article.)

In order to demonstrate that our honeycomb gold discs with subsequent FIB milling enable high-resolution structure determination, especially given the gold redeposition described above, we sought to reconstruct an average of the prokaryotic 70S ribosome by sub-tomogram averaging (STA). Ribosomes are the most tractable target for STA: they are large (2.5 MDa) and abundant (thousands per cell). Using current state-of-the-art cryo-EM image-processing tools (see Materials and Methods, Fig. S4) we reference-free averaged 15,596 sub-tomograms from 23 tomograms to generate a structure of the 70S ribosome, at 6.7 Å nominal resolution (Nyquist 5.2 Å) (Fig. 6 a, b, h). At this resolution, protein α-helices are clearly visible as tubular densities and RNA is well resolved (Fig. 6 c-f). Unsurprisingly, local resolution estimation indicates that peripheral parts of the rRNA are not as well resolved as the core (Fig. 6 g). For instance, the L1 stalk displays local resolution worse than 10 Å, likely owing to its flexibility. While preliminary, these results show clearly that the lamellae generated by FIB milling our honeycomb discs can be effectively interrogated for sub-nanometer resolution structural information.

## 3 Discussion

We have developed an approach of fabricating custom honeycomb gold supports to trap bacteria in a vertical orientation which, when combined with FIB milling, allow the generation of lamellae containing many vertical cross sections through bacteria. In future, we will use this technology for *in situ* structural studies of the Z-ring and the FtsZ-controlled divisome, the multi-protein complex that synthesises and remodels the cell wall, causing cell constriction. In order to achieve this, these supports will need to be used in combination with other technological and methodological developments. Since the advent of fast tilt series collection strategies such as PACEtomo (Eisenstein et al., 2023), the FIB milling step is now the main bottleneck in the workflow and higher milling throughput will be necessary for structure determination of proteins less abundant than the ribosomes averaged in this work. This could be achieved by using the newest generation of milling instruments (Berger et al., 2023). Plasma FIBs, for example, can mill at higher beam currents than currently available gallium ion sources, and so can be used to speed up at least the early rough milling steps, for which damage due to milling is less of a concern, since these early steps are distant (on the order of microns) from the eventual lamella. Fully automated milling will also increase the throughput, and autoloader systems can be used to reduce specimen handling steps and to minimise ice contamination on milled lamellae (Buckley et al., 2020; Cleeve et al., 2023; Klumpe et al., 2021; Zachs et al., 2020).

One of the drawbacks of using gold in the support is the redeposition of sputtered gold onto the surface of the lamellae. The loss of information and high contrast artefacts that this causes may be a barrier to achieving high resolution with subtomogram averaging. While in this instance we were able to reconstruct the ribosome *in situ* to 6.7 Å, it should be noted that the ribosome is very large compared to FtsZ. Moving forward we suggest that improvements in instrumentation to prevent redeposition of the ion-sputtered material in general, and gold in particular, will also be of great interest. For example, changes to the sample holder and chamber geometry so that the flight path of debris does not end up back on the specimen (e.g. sample supported by the rim of the autogrid only, in a similar manner to that in a TEM) may help to reduce the level of gold contamination. The newest cryo-plasma-FIB instruments also have improved vacuum and cryoshielding which may also reduce ice contamination (Berger et al., 2023) and improved cryo shields can be retrofitted to some older instruments (Tacke et al., 2021).

Another major challenge is the identification of proteins of interest within the crowded and complex cellular environment. The reduced movement during imaging in the TEM afforded by this specimen support will help with this as it will improve at least initial tomogram quality. While CLEM approaches can be used to localise to a region of the cell, they do not currently have the capabilities to localise and correlate individual proteins (fluorophores), although from a theoretical perspective this should be feasible with technological improvements (Dahlberg et al., 2020; Moser et al., 2019; Phillips et al., 2020; Sartori et al., 2007; Tuijtel et al., 2019). An alternative to CLEM is to use labels that are directly visible in the TEM. While approaches using cloneable, electron dense tags such as ferritin (Wang et al., 2011) or metallothionein (Mercogliano and DeRosier, 2007), or DNA origami (Silvester et al., 2021) as labels have been demonstrated, all of these methods perturb the biological system beyond what is likely acceptable to gain reliable biological insights in most cases. As shown here, filamentous proteins such as FtsZ are easy to recognise in tomograms without labelling. Labelling approaches will probably be necessary to identify divisomes, although it may also be possible to identify them in tomograms directly, based on their connectedness with the Z-ring and the reduced background signal in the periplasm.

While the discs produced for this study have a specific design to match the bacterial specimens of interest, the general nature of the fabrication method means that this approach could be adapted to answer other biological questions, for any situation where it would be useful to have a well-defined geometry for vitrification, milling and imaging. Furthermore, we feel it is also important to point out that single particles will likely orient randomly in the honeycomb wells due to the change in ratio of surface to volume and decreased air–water interface. Therefore, subsequent milling and imaging could overcome preferred orientation problems for those where it exists using standard single particle sample preparation approaches (Fig. S5) and the milling would remove those particles adsorbed to the air water interface, which can cause preferred orientation and denaturation (Glaeser, 2018; Noble et al., 2018). This will depend on the ability to make the milled samples sufficiently thin without too much damage to the sample by the milling procedure. Recent attempts to characterise the damage layer suggest that it may extend up to 60 nm from the lamella surface (Berger et al., 2023; Lucas and Grigorieff, 2023; Yang et al., 2023). The single particle use of the honeycomb grids will also require the ability to mill with high enough throughput to support the collection of the hundreds to thousands of images required for single particle analysis.

We envision a range of similar specimen supports, tailored to imaging molecules in a wide range of cellular and molecular specimens, will now be straightforward to create.

## 4 Materials & methods

### 4.1 Disc manufacture

Honeycomb discs were manufactured using 4-inch ⟨100⟩ silicon wafers (University wafer) as a base. First a 40–50 nm layer of copper was evaporated onto the wafer using an electron beam evaporator (Moorfield Minilab 080). Immediately after removal from the vacuum chamber of the evaporator, the wafer was transferred to a spin coater (Cee Apergee 450). Approximately 4 mL of omnicoat (Kayaku Advanced Materials) was static dispensed onto the wafer before spin coating using a 2-step program (spread step: 500 rpm, acceleration 100 rpm/s, 5 s; coat step: 3000 rpm, acceleration 300 rpm/s, 30 s). The wafer was incubated on a hotplate at 200 °C for 60 s to soft bake the omnicoat, removed and allowed to cool to room temperature before spin coating with SU-8 2005 (Kayaku Advanced Materials). Approximately 4 mL of resist was statically dispensed onto the wafer and spin coated using a 2-step program (spread step: 500 rpm, acceleration 100 rpm/s, 6 s; coat step: 4000 rpm, acceleration 300 rpm/s, 40 s). The wafer was incubated on a hotplate at 95 °C for 2 mins to soft bake the SU-8, then left to cool at room temperature for 2 min before transfer to a mask aligner (Neutronix-Quintel Q4000). The wafer was raised to vacuum contact with the lithography mask (Fig S1, ordered from Compugraphics) and UV exposed for 15 s at a dose rate of 9.0 mW/cm2. This was followed by a post-exposure bake at 95 °C for 2 min and the wafer was allowed to cool to room temperature. The SU-8 was developed by immersion in propylene glycol monomethyl ether acetate for 2 min with gentle agitation, washed by immersion in isopropanol, and dried with a stream of dry nitrogen gas. Finally, the omnicoat was developed by immersion in Microposit MF 319 developer (Kayaku Advanced Materials) for 15 s with very gentle agitation, then washed by immersion in 3 successive water baths. The wafer was dried with a stream of dry nitrogen gas, left in a laminar flow hood for ~ 30 min to allow any remaining moisture to dry and hard baked in an oven at 70 °C for 10 mins.

The patterned wafer was electroplated with gold using a Yamamoto 4-inch silicon wafer plating set with MetGold ECF33B cyanide-free plating solution (Metalor Advanced Coatings). The wafer was cleaned using a UVO cleaner (Jelight, model 42 series) for 4 min, assembled into the cathode holder, pre-heated to 60 °C in a dry oven for a minimum of 20 min and the gold solution pre-heated to the plating temperature of 54 °C. The wafer was plated for 15 min with the voltage continuously monitored and adjusted to give a current of 0.28 A, which should result in a nominal depth of plating of 4 µm for the design used. The wafer was transferred to a water bath at 54 °C and gradually cooled to room temperature by the addition of cold water. The cathode holder was disassembled and the wafer further washed by sequential immersion in 2 water baths and dried with a stream of dry nitrogen gas.

Before any further processing, the wafer was cut into 13 separate chips, each chip containing 40 discs. The sheet of discs was loosened around the edges of the chips and then was released from the wafer by an overnight piranha etch (3:1 sulphuric acid: hydrogen peroxide). To ensure all copper and photoresist was removed, the released film was subjected to a second piranha etch in fresh solution for a further 4 h. This was followed by 3 sequential water washes and the discs were then allowed to air dry. Finally, the discs were inspected by SEM to check for defects and confirm the diameter of the wells, before separation by cutting the joints holding supports together manually with a scalpel.

### 4.2 Bacterial culture and specimen preparation

For FtsZ(D212A) overexpression, C41(DE3) *E. coli* cells were transformed with plasmid pMZ120 (Szwedziak et al., 2014) and cultured in M9 minimal media supplemented with 0.4 % glycerol at 37 °C until approximately OD600 0.4. 10 mL of culture were centrifuged and washed in PBS before a 10-minute incubation with 200 µL of PBS pre-mixed with 1 µL CellBrite Fix 488 (Biotium). The dye was washed out by centrifugation and the pellet mixed with 200 µL of M9 supplemented with 0.1 % arabinose. The honeycomb discs were glow discharged at 30 mA for 2 min on each side. 30 µL of the concentrated culture was added to the surface of each disc to be vitrified and centrifuged for 1 min in a custom-made plate holder (Fig S2) in a table-top swinging bucket centrifuge at 3000 rpm (~1800 g). A further 10 µL was added to the disc, pipetting across the top surface to dislodge cells blocking entrance to the wells, and the centrifugation step repeated. A final 10 µL was added as above and the discs were subjected to a third and final centrifugation step. The surface of the disc was washed with arabinose supplemented M9, a 50 µL drop of fresh media added to the surface and the discs were incubated inside a humid chamber at 37 °C for approximately 1 h. The discs were removed from the incubator, front- (hole) side blotted and plunge frozen in liquid ethane using a manual plunger. For tomography of native FtsZ, C41(DE3) *E. coli* cells were cultured in LB at 37 °C until OD 0.5–0.6, washed into PBS and incubated with membrane dye as above.

### 4.3 FIB milling

Lamellae were milled on either a Scios Dual Beam FIB-SEM (Thermofisher Scientific) or a Crossbeam 550 (Zeiss), both equipped with a PP3010 cryo-stage and loading system (Quorum Technologies). Prior to milling, the discs were coated with an organo-platinum layer for 30–45 s using the Gas Injection System (GIS). Bulk milling and expansion joint milling were performed at 30 keV, 5–15nA using rectangular milling patterns and all subsequent steps were performed using cross-sectional milling. The milling currents were gradually reduced as the lamellae were thinned till the final polishing step at 30–100 pA. Once the lamellae were below ~ 1 µm thickness, milling was performed with an under/over tilt of 0.5-1°, until the final polish, which was performed at the original milling angle.

### 4.4 Cryo fluorescence microscopy (CLEM)

Milled lamellae were fluorescently imaged on a LSM900 Axio Imager Z2 Airyscan confocal microscope (Zeiss), equipped with a cryo-stage for imaging at liquid nitrogen temperature (Linkam CMS196V3). Z-stacks were collected using a 100x air objective (LD EC Epiplan-Nerofluar 100x/0.75 DIC) using airyscan, with a pixel sampling of 0.079 µm x 0.079 µm x 0.210 µm and a pixel time of 27.3 µs. The raw stacks were 3D processed (pixel reassignment and deconvolution) using the airyscan processing routine within Zeiss ZEN software with the auto function to determine the strength of the Wiener filter. Z stacks were imported into Fiji software (Schindelin et al., 2012) and a maximum intensity Z-projection generated.

Pre-screening of grids was performed in the same cryo-confocal instrument as above, imaging in widefield mode. To estimate well occupancy, the 100x objective was used and images were either collected at random selected points across the grid, or tiling was used over large areas.

### 4.5 Electron microscopy

Lamellae were imaged using a Titan Krios electron microscope (Thermofisher Scientific) equipped with a Gatan imaging filter and K3 direct electron detector (Gatan). Tilt series were collected using SerialEM (Mastronarde, 2005), either using the SerialEM batch tomography setup or PACE-tomo (Eisenstein et al., 2023). Tilt series were collected from a start tilt of –10° (to compensate for the tilt of the lamella, which are milled at an angle of approximately 10° to the disc and loaded with a rotation of approximately –90° between the milling orientation and the Krios autoloader) in a dose symmetric manner (Hagen et al., 2017), with an increment of 2° and a tilt range of ± 50–60° from the start tilt. Each tilt series had a total fluence of approximately 150 e ^−^ /Å^2^, distributed evenly across the tilt series and each tilt was collected in 5–6 frames. The pixel size was either 2.1 Å or 2.7 Å depending on the particular data set.

### 4.6 Tomogram reconstruction

Frames were aligned using IMOD (version 4.10.25) alignframes (Kremer et al., 1996). Tilt series were aligned using patch tracking and reconstructed by back projection, both using etomo, part of the IMOD package (Kremer et al., 1996). Tomograms were denoised using cryo-CARE (Buchholz et al., 2019). For training of the cryoCARE noise2noise model, tilt series were produced using odd/even aligned frames and then reconstructed using the alignments generated for all frames. For each tomogram to be denoised, the model was trained separately on just the odd/even frame generated tomograms of the tomogram in question. Segmentation and modelling were done manually in IMOD. Membranes were modelled by drawing contours every 20 slices. As it was not always possible to distinguish individual filaments in Z and determine their central slice, contours were drawn on every slice where filaments were visible, so each filament is represented by multiple contours, rather than a single line.

In order to analyse the orientations of the cells in the wells, we annotated the surfaces of lamellae and the cell envelope with control points. By least-squares fitting linear, planar, and elliptical models to these control points, the relative orientations of each lamella and cell axis were estimated.

### 4.7 Comparison of motion between the custom supports and conventional lamellae

*E. coli* (C41(DE3)) cells were cultured to mid log phase as above, concentrated 50x by centrifugation and applied to 200 mesh EM grids with a R2/2 holey carbon support film (Quantifoil). The grids were back blotted and plunge frozen in liquid ethane using a manual plunger. Lamella were milled using a Crossbeam 550 (Zeiss), equipped with a PP3010 cryo-stage and loading system (Quorum Technologies). Prior to milling, the grids were sputtered with a conductive platinum layer (5 mA for 45 s), then coated with an organo-platinum layer for 30–45 s using the Gas Injection System (GIS). Milling was performed in a similar manner as for the custom discs above, with the following exceptions. Bulk milling and expansion joints were milled at 30 keV 1nA, stepping down to 50 pA for the final polish. No under/overtilting was used and the lamellae were milled 8–10 µm wide (as opposed to 25–30 µm for the custom supports).

In a single Krios session, both honeycomb discs and regular milled grids were loaded. Tilt series were collected at 2.7 Å/pixel over 6 subframes as described above. The frames were motion corrected using Warp (Tegunov and Cramer, 2019), and the mean motion per frame used to generate the violin plot in Fig. 3f.

### 4.8 Sub-tomogram averaging

The following procedure is schematised in supplementary Figure S3. 143 tilt series (collected at 2.7 Å/px) were automatically reconstructed at bin 5 using a custom script based around IMOD programs (Kremer et al., 1996), using alignframes for frame alignment, cross-correlation based tilt series alignment and reconstruction by back projection. The resulting tomograms were inspected to determine lamellae thickness and 83 with a thickness above 200 nm were discarded. The remaining 60 tomograms (thicknesses ranging from 60 to 200 nm) were selected, and the tilt series were manually realigned by patch tracking in etomo, part of IMOD (Kremer et al., 1996). Defocus estimations were calculated in Warp 1.0.9 (Tegunov and Cramer, 2019). Tomograms for particle picking were reconstructed in RELION 4.0 (Zivanov et al., 2022) at 8 × binning (21.3 Å/px) with CTF demodulation. For further processing, we selected 23 tomograms out of our initial shortlist of 60 whose alignments were visually superior and whose reconstructions captured the greatest biological detail: membranes and ribosomes were clearly visible. Tomograms in which there was cracked ice or heavy gold or ice contamination were discarded. We also discarded any tomograms that were collected from horizontally oriented cells in the ice layer on the top of the supports.

On a previous dataset (collected at 2.13 Å/px), 1,280 suspected ribosomes were picked by hand from 33 representative slices across 8 tomograms. These particles were used to train a crYOLO model (Wagner et al., 2019). This model was then allowed to pick on our 23 tomograms, producing ~ 43,500 picks. These particles were inspected in the tomograms that they originated from, and those that were obviously not ribosomes (because, for instance, they lay outside any cell) were manually discarded. This left ~ 39,000 particles.

The particle coordinates were supplied to Warp for sub-tomogram generation, using starfile (Burt et al., 2021). Sub-tomograms were first extracted at 4 × binning (10.6 Å/px). These sub-tomograms were classified in RELION, with the aim to filter out particles that were not ribosomes. It appeared that almost all particles were in fact ribosomes, and they averaged to a resolution of 21 Å (Nyquist sampling limit at 4 × binning), so the same coordinates were used to generate sub-tomograms at 2 × binning (5.3 Å). Classifying these sub-tomograms into 8 classes with alignment in RELION yielded 3 sensible classes, comprising 40 % of the particles (~15,500). Refining the poses of these 15,500 particles produced an 11 Å–resolution map (Nyquist at 2 × binning). Finally, unbinned sub-tomograms were extracted for these ~ 15,500 particles. Refinement generated a 9.3 Å map. From here, M (Tegunov and Cramer, 2019) was used to refine geometric parameters and defocus. M produced a 6.7 Å–resolution map, clearly resolving protein secondary structure.

## Supplementary Material

Supplementary data to this article can be found online at https://doi.org/10.1016/j.jsb.2024.108097.

Appendix A. Supplementary data

## Data Availability

Data will be made available on request. A ribosome map acquired by sub-tomogram averaging is available as EMDB entry 18826.
