# Supplementary material for: Honeycomb gold specimen supports enabling orthogonal focussed ion beam-milling of elongated cells for cryo-ET
Source: J Struct Biol. Author manuscript; Available in PMC 2024 Jul 22. (PMC7616276; doi:10.1016/j.jsb.2024.108097)
Supplement: Appendix A. Supplementary data [file EMS197563-supplement-Appendix_A__Supplementary_data.zip › Supplementary Information JL 28 3 24.docx]

**SUPPLEMENTARY INFORMATION**

**Honeycomb gold specimen supports enabling orthogonal
focussed ion beam-milling of elongated cells for cryo-ET**

Victoria L. Hale, James Hooker, Christopher J Russo, Jan Löwe

**Contents:**

Figure S1 – S5

**
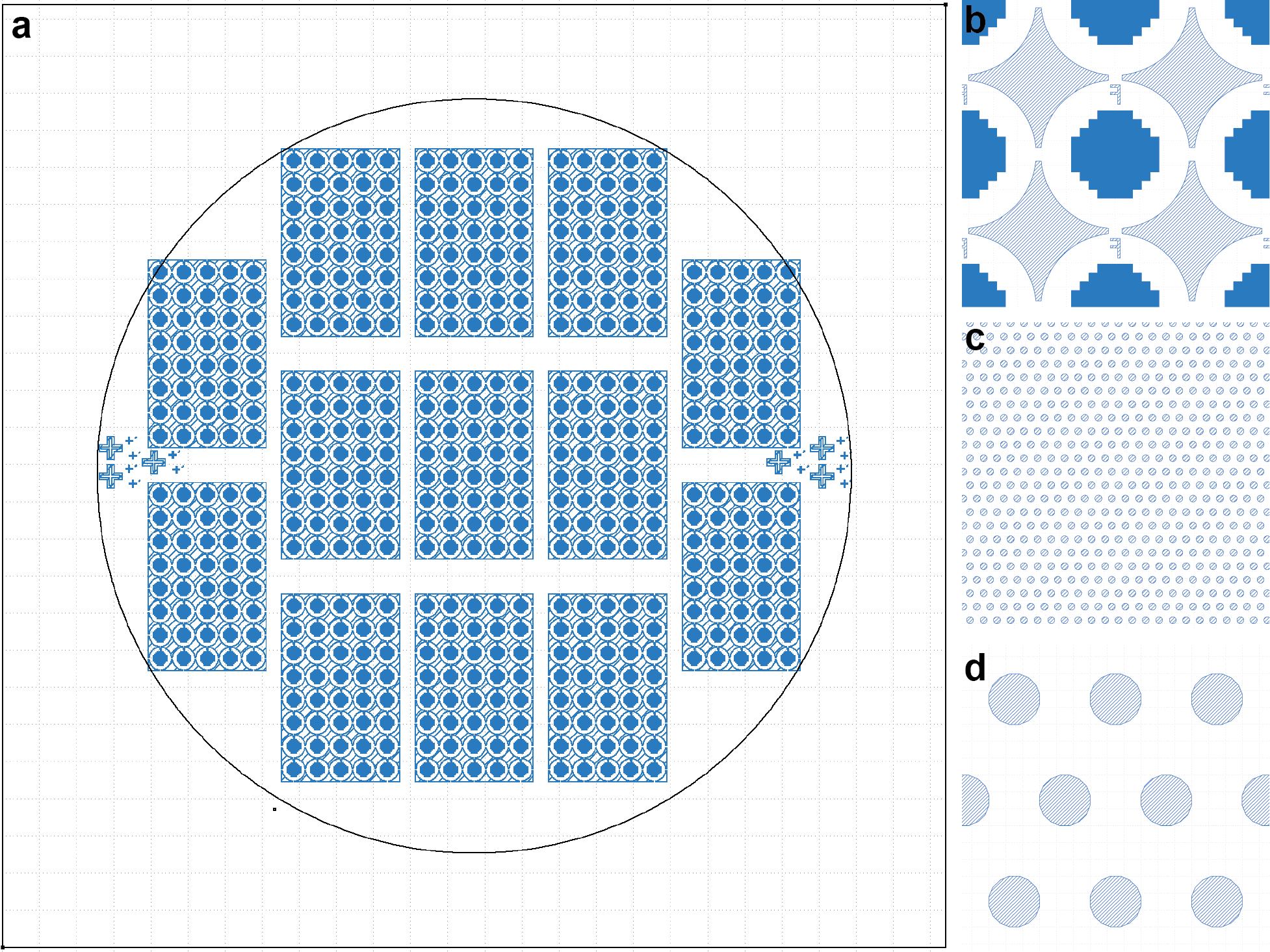
**

**Figure S1: Mask design**. The mask design used in this study. The whole mask is depicted in (a), (b) shows the central region at the disc level, and (c, d) show the well level at increasing zoom factors. Filled blue areas are clear on the printed mask, all white areas are opaque. Small features (the circles) appear as solid blocks in (a) and (b). The black square and circle in (a) show the mask edge and approximate position of the silicon wafer respectively. The mask file is supplied in Supplementary Data File D1.


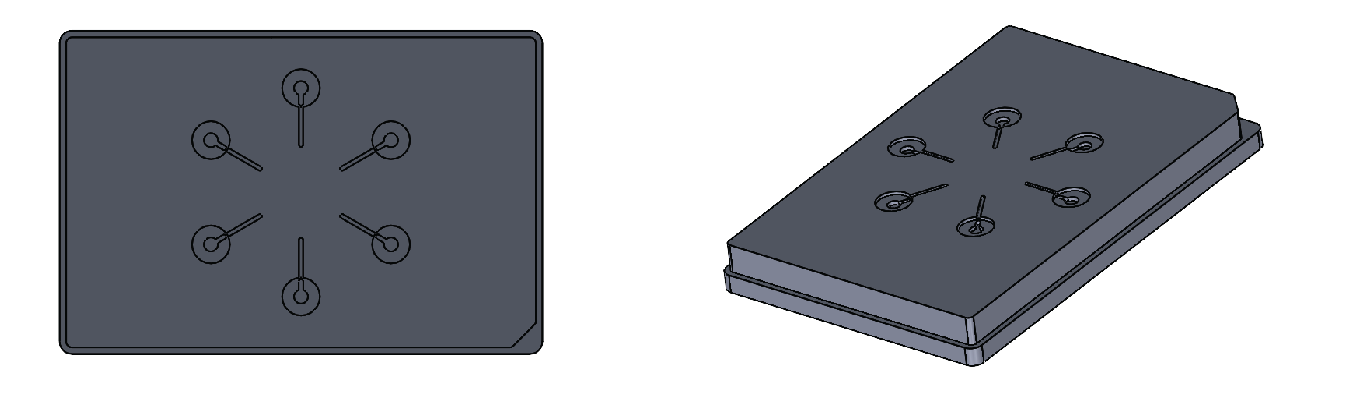


**Figure S2: Centrifuge plate for loading the honeycomb discs**. Custom-designed holder for centrifuging the cells into the honeycomb wells. The plate has space for 6 discs which sit inside a depression inside a larger well which contains liquid. The long notch allows washing of the disc surface by pipetting across. For centrifugation, the holder is placed into swinging-out centrifuge bucket so the centrifugal force is normal to the holder and disc surfaces.

**
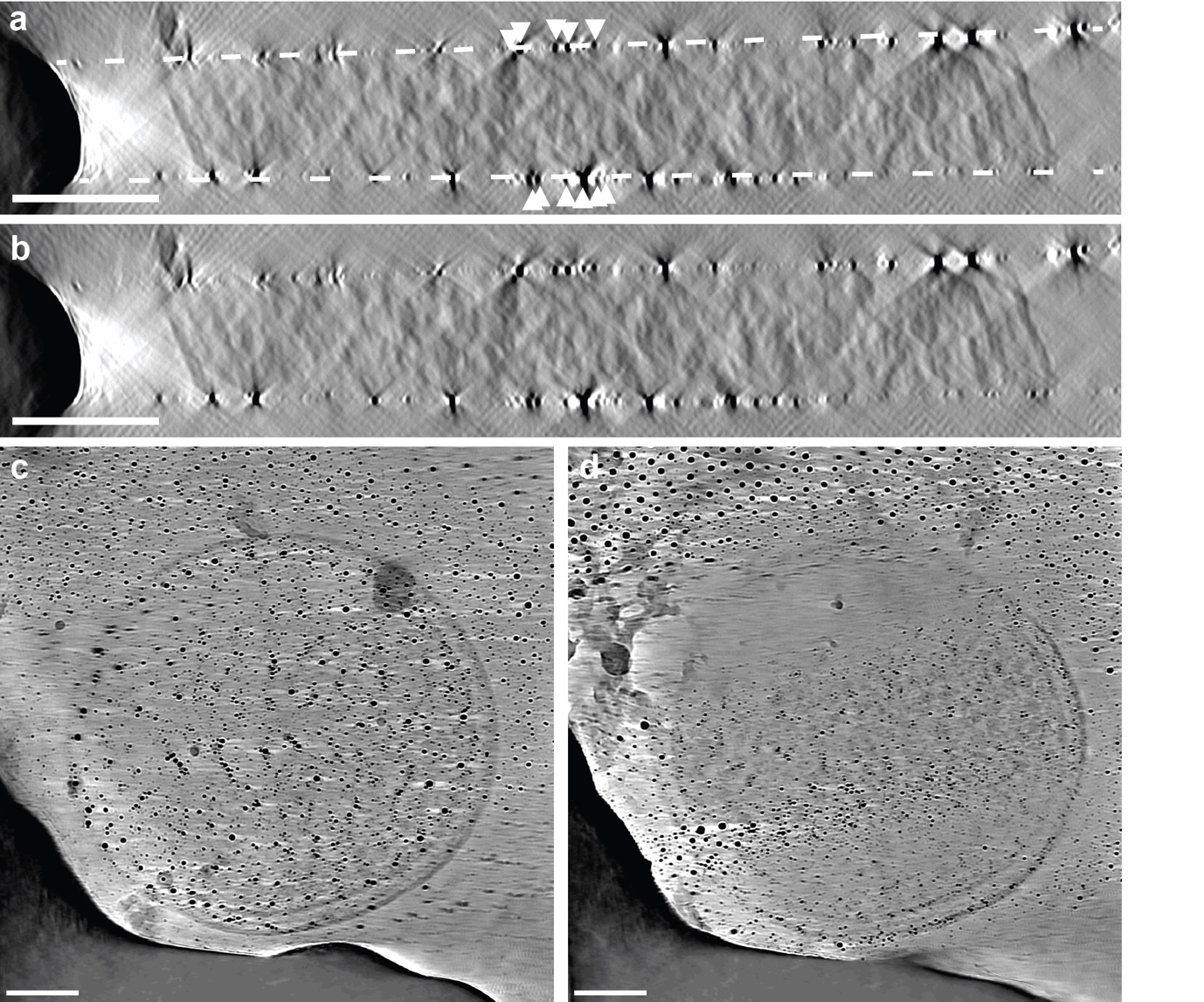
**

**Figure S3: Surface gold.** (a, b) XZ section of the tomogram depicted in Fig. 4a, shown with (a) and without (b) annotation. Dashed lines indicate the two surfaces of the lamella in (a). Gold particles are present on each surface of the lamella as indicated with arrow heads in the central portion of (a). Lamella thickness 80-100 nm. (c, d) Top and bottom surfaces of the same tomogram. The sum of 50 slices (approx. 21 nm) has been shown to include the gold on the same plane. Scale bars 100 nm.

**
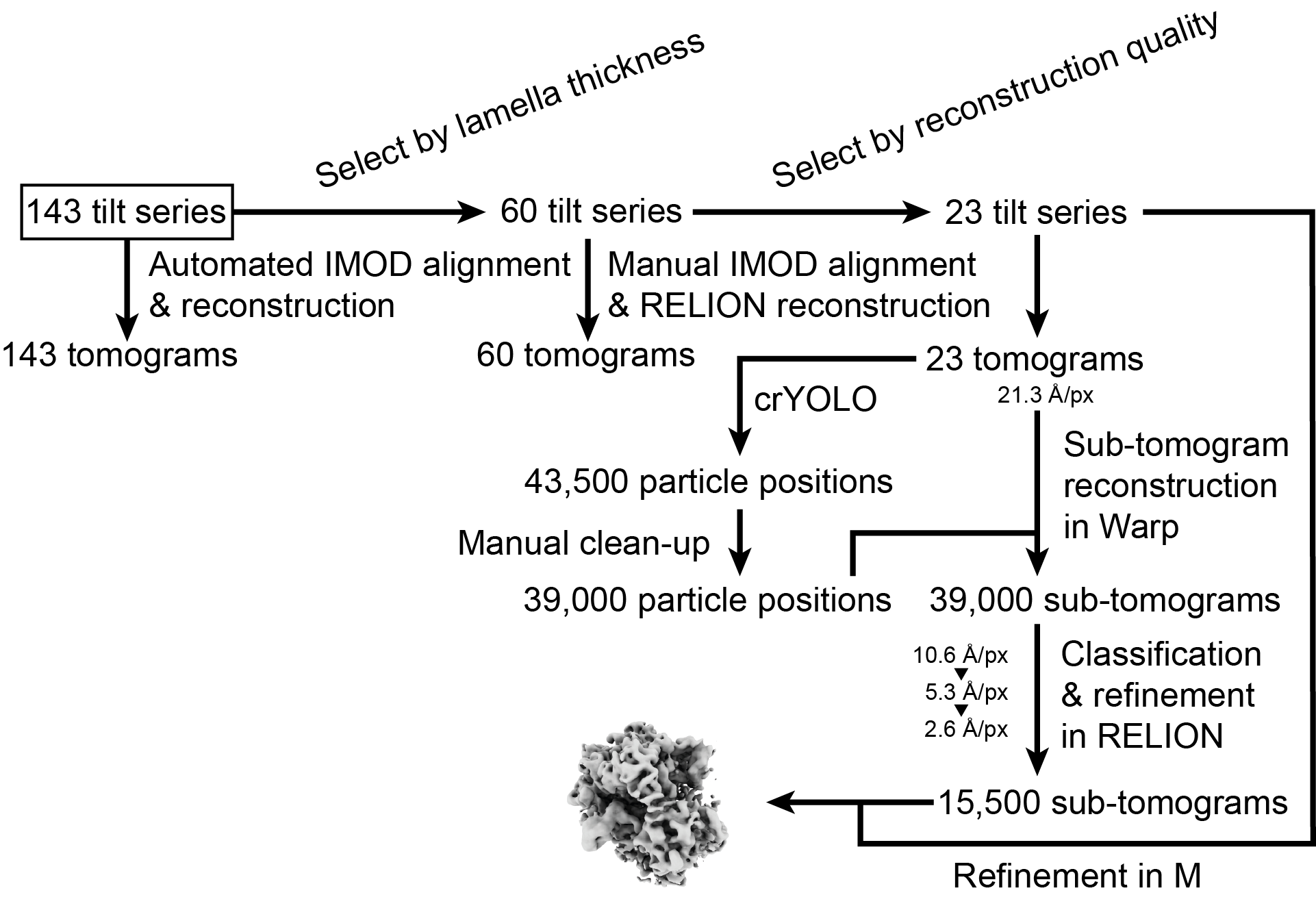
**

**Figure S4: Sub-tomogram averaging workflow.** An outline of our averaging workflow from a dataset of 143 tilt series (top left) to a 6.7 Å map of the *E. coli* 70S ribosome (bottom). The dataset was progressively filtered according first to local lamella thickness and then to the quality of the tomographic reconstruction. IMOD was used to align tilt series, RELION to reconstruct tomograms for picking, crYOLO to pick particles, Warp to generate sub-tomograms, RELION and M for particle pose optimisation and averaging.


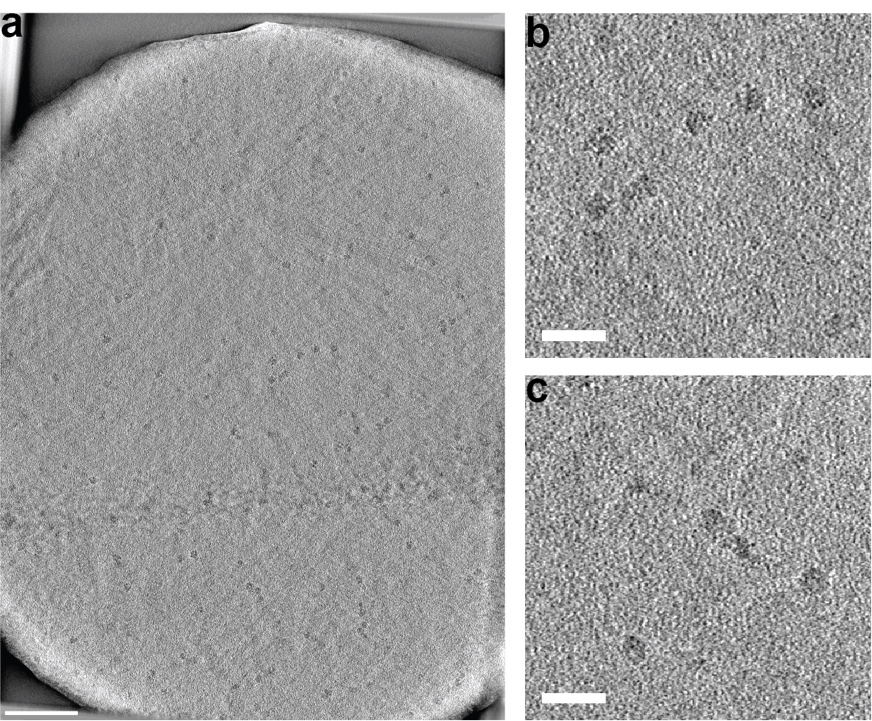


**Figure S5: Milled honeycomb discs with ribosomes.** The honeycomb discs were loaded with ribosomes, plunge frozen, FIB milled and tilt series were collected. Tomogram slices of the whole well (a) and smaller regions (b, c) are shown. Scale bars 200 nm (a), 50 nm (b, c).
